# Thermal Conductivity of FLiNaK in a Molten State

**DOI:** 10.3390/ma15165603

**Published:** 2022-08-15

**Authors:** Alexey Rudenko, Alexander Redkin, Evgeniya Il’ina, Svetlana Pershina, Peter Mushnikov, Yuriy Zaikov, Sergey Kumkov, Yalan Liu, Weiqun Shi

**Affiliations:** 1 Institute of High Temperature Electrochemistry, Ural Branch of the Russian Academy of Sciences, Akademicheskaya Str., 20, 620137 Yekaterinburg, Russia; 2 Institute of Mathematics and Mechanics, Ural Branch of the Russian Academy of Sciences, St. S. Kovalevskaya 16, 620990 Yekaterinburg, Russia; 3 Institute of High Energy Physics, Chinese Academy of Sciences, No. 19B Yuquan Road, Shijingshan District, Beijing 100049, China

**Keywords:** FLiNaK, molten salts, thermal diffusivity, heat capacity, thermal conductivity

## Abstract

Although the thermal conductivity of molten salt mixtures is of interest for many potential technological applications, precise values are often hard to obtain. In this study, the thermal diffusivity of FliNaK was studied in a molten state using the laser flash method and found to be very slightly dependent on temperature. The heat capacity of FliNaK was measured using the DSC method. There was a minor difference between our results and data from the literature. From calculations based on thermal diffusivity, density and heat capacity values, thermal conductivity was shown to decrease with temperature.

## 1. Introduction

The systematic study of the properties of molten salts, which began at the beginning of the 20th century, was initially focused on investigating density and electrical conductivity. At that time, molten salts were being considered as electrolytes for the production of metals. With the advent of the Molten Salt Reactor project (MSR) in the USA in 1950s, the thermal properties of molten salts became a topic of interest. Since then, a number of methods have been applied for conducting thermal conductivity measurements. However, due to certain disadvantages inherent in all these methods, the obtained results varied. Methods that use direct detection of the temperature difference at the boundaries of the molten salt layer generally give a positive temperature dependence. Conversely, more sophisticated techniques such as the laser flash method tend to show a negative thermal conductivity temperature dependence. A comprehensive comparison of all known methods was given in a review [1]. The authors distinguished the methods into two groups: reliable and non-reliable. Here, the main classification criterion was temperature dependence; the tendency for direct methods to give a strong positive temperature dependence was considered to be due to significant heat losses arising from convection and other factors. However, the fact that similar results are obtained from all methods at temperatures close to the melting point permits the combination of thermal conductivity results [2]. This approach supports the conclusion that molar thermal conductivity is a constant for certain types of salts, allowing the thermal conductivity of mixtures to be estimated across broad temperature and concentration intervals.

Molten eutectic mixtures of lithium, sodium and potassium fluorides (0.465 LiF–0.115 NaF–0.42 KF) are of interest for possible use as media in molten salt reactors. In comparison with other alkali fluoride mixtures, such mixtures exhibit significantly lower melting temperatures to allow the working temperature of process to be reduced below 900 K. However, in order to develop the technological process, thermophysical properties such as heat capacity and thermal conductivity are required.

**Figure 1:**
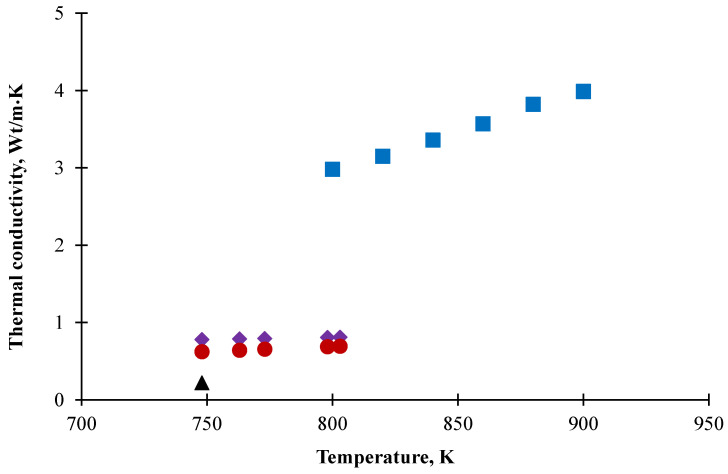
FLiNaK thermal conductivity data obtained by Khokhlov (⯁), An (●), Ueki (▲) and Ewing (■).

The thermal conductivity of FLiNaK was measured for the first time by Ewing using the parallel plates method [3]. However, the obtained results were far above values characteristic for molten salts. Further investigations of FLiNaK’s thermal conductivity were conducted using the coaxial cylinder method to produce more reliable values [4,5]. The laser flash method was used in subsequent measurements [6] to yield results lower than the previous ones. The uncertainty of thermal diffusivity measurements in this work was about 25%, placing these results in the non-reliable class. The results of the latest measurements carried out by Ueki are also much lower than all previous results [7]. As can be seen from the old data given in Figure 1, the results demonstrate a significant variety of values. Although fresh measurements performed this year are closer to each other, they still differ by temperature dependence [8,9]. Thus, reliable data on thermal conductivity of molten FLiNaK are needed for modeling of real objects based on this mixture.

The heat capacity of FLiNaK has been measured byKhokhlov [5], An [6] and Janz [10]. While the results obtained by the first two works are close to each other, those obtained by An are slightly higher. The density of molten FLiNaK is well-established [11,12]. Data on density are in a good agreement within 3%; heat capacity results differ less (10%), but thermal conductivity values for FliNaK are different by order and have to be precise.

The objective of the present study is to reduce uncertainty in thermal conductivity values. This becomes possible due to the development of new scientific equipment for thermo-physical investigations based on the laser flash method.

## 2. Materials and Methods

### 2.1. Preparation of Samples

All manipulations of selected species were carried out in a glove box under a controlled inert argon atmosphere (SPEKS GB-02M). The moisture and oxygen content did not exceed 1 ppm. The samples of lithium fluoride (reagent grade) and beryllium fluoride (reagent grade) were mixed in the required ratio to an accuracy of 1 mg in order to obtain 40–50 g of the melt. The salt mixture was loaded in a glassy carbon crucible into a nickel retort, placed in a resistance furnace and heated to a temperature of 973–1023 K. After holding in a molten state for at least three hours, the samples were placed in a special nickel container and taken for chemical analysis.

The samples containing potassium chloride were prepared from salt purified by zone melting. For the KNO_3_-NaNO_3_ samples, solidified crystals obtained from a melted chemically pure reagent were used.

Samples were taken from the melt of the required composition using a spoon-shaped nickel sampler in a box under a controlled argon atmosphere (oxygen less than 5 ppm, moisture less than 1 ppm). Following solidification, sample droplets weighed about 0.4–0.7 g. To obtain a total weight of about 1.5 g, several sample droplets were placed in a nickel container and heated in a furnace above melting point. After melting, the absence of gas bubbles at the bottom was visually monitored. If bubbles occurred, they were driven out by stirring with a nickel wire. Then, the container was removed from the furnace and closed with a lid while still hot. Then, the entire container assembly was, again, heated to a temperature above melting point. After cooling, they were taken out of the box in a sealed container, from which they were taken out immediately before welding. After welding by an argon-arc apparatus, the samples were again brought into the box and heated above the melting temperature. After cooling, the samples were labeled and packed into containers.

### 2.2. Laser Flash Method

At present, there is no universal wide-approved technique for thermal diffusivity and thermal conductivity measurements of molten salts. All techniques have some disadvantages that affect results. Different temperature dependence is a result of different unaccounted factors influence.

Thermal diffusivity was measured using the laser flash technique developed for the molten media [13,14,15,16]. The measurements were carried out using a Netzsch Laser Flash 267 device. The sample was placed in a holder inside a high-temperature electric furnace. Radiation was generated by a halogen lamp across the entire visible wavelength.

**Figure 2:**
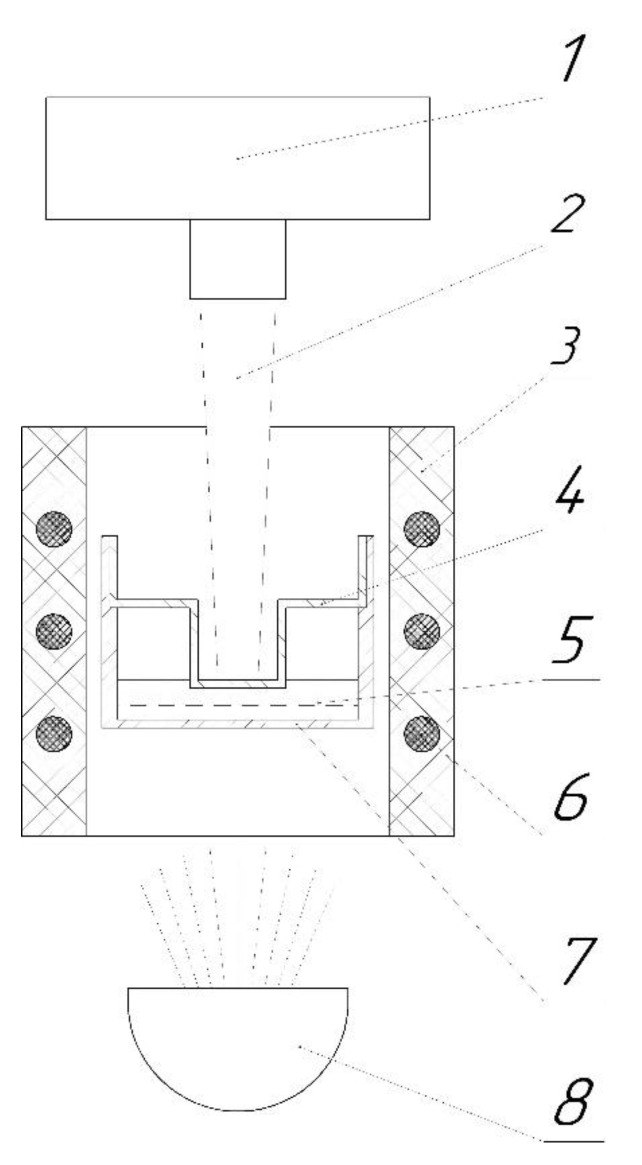
Measuring cell scheme for the laser flash method: 1—IR Detector; 2—IR Beam; 3—Furnace; 4—Lid; 5—Sample; 6—Heater; 7—Crucible; 8—Halogen Lamp.

The change in the temperature of the upper surface of the sample was recorded using an IR detector based on indium antimonide (InSb) cooled with liquid nitrogen, having an area of about 3 mm in diameter. The laser pulse amplitude was recorded by a separate detector. The design of the setup allows samples to be measured across a wide temperature range from room temperature to 1250 °C under an inert atmosphere (Ar, He) or in a vacuum. The temperature of the holder was measured using a thermocouple installed in the immediate vicinity of the sample (1–2 mm). The cell measurement scheme is given in Figure 2. The cell was made of nickel alloy NP2. The cell material was selected on the basis of its physical and chemical properties. The thermal diffusivity of the cell material was investigated in separate experiments. The typical thicknesses of the crucible bottom and insert were about 0.5 mm at a melt layer of 1–2 mm. The side walls and lid of the crucible had a thickness of around 0.5 mm and outer diameters of 17 mm and 5 mm, respectively.

In order to exclude all side effects, the cell was calibrated using water; the calibration was then verified on molten KNO_3_-NaNO_3_ mixture.

During the high-temperature measurements, the NP2 alloy was found to retain a bright metallic luster; no darkening was observed even following prolonged high-temperature experiments. In order to increase the degree of blackness, the surfaces of the cell were processed with emery paper and covered with graphite spray (thickness ~5 μm). Following the experiment, the graphite was easily removed with alcohol; the NP2 material does not interact with carbon. The cell shape did not change after high-temperature experiments.

### 2.3. DSC Method

Heat capacity investigations were carried out using an STA 449F1 Jupiter^®^ synchronous thermal analyzer (NETZSCH, Selb, Germany), which allows changes in the sample mass and differential scanning calorimetry (DSC) curve to be recorded synchronously. The experimental setup provides high measurement accuracy of the specified parameters: temperature (±1.5 K); mass (<10^−6^ g); baseline reproducibility (±2.5 mW); enthalpy (±3%). Single-crystal sapphire was used for sensitivity calibration. The measurements were carried out under the following conditions: temperature range: heating—308–773 K, cooling—773–473 K; heating rate—10 K/min; atmosphere—pure Ar (special purity); Pt-Rh crucibles with lids. The test samples with a weight of about 10 mg were preheated to 1023 K and kept for 1 h in a muffle furnace placed in an Ar glove box. In order to determine the heat capacity of the sample under study, the thermal behavior of the initial system was first determined when both crucibles were empty to form the baseline. Then, using the same experimental conditions (heating rate, crucibles, atmosphere, etc.), measurements were made for a *DSC* sapphire reference sample having a mass m and a known heat capacity *C_p_*. The third measurement was performed for the test sample having a known mass m. The specific heat capacity of the test sample (*C_p_*) was calculated by the formula

(1) $$C_{p}=\frac{m_{st}}{m_{sample}}\cdot \frac{DSC_{sample}-DSC_{base}}{DSC_{st}-DSC_{base}}\cdot C_{p\;st}$$

All measurements were carried out under the same conditions. The calculations were performed using the NETZSCH Proteus program. The standard error of temperature measurement is 5 K. Taking the three-stage measurement process into account, the standard error of the heat capacity measurement is 5%. When studying such complex objects as molten salts, in which significant changes in their shape can occur following melting, the error can be estimated at 10%. The measurements and calibrations were carried out under conditions of heating and cooling. The experiments complied with the conditions of ethical approval for intervention studies involving animals or humans, and other factors.

## 3. Results

### 3.1. Thermal Diffusivity

In order to exclude the influence of possible measurement side effects, the thermal diffusivity of the molten eutectic mixture of KNO_3_-NaNO_3_ was measured after calibrating the cell using water. The results are given in Table 1. The thermal conductivity was calculated using density and heat capacity data from the literature. The results are shown in Table 1 alongside data obtained by other authors. There is good agreement with the literature data.

**Figure 3:**
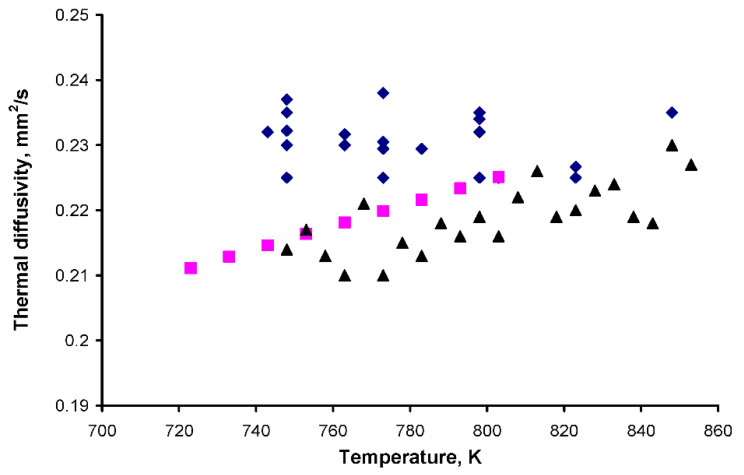
Thermal diffusivity data for FLiNaK: Our result (⯁), Kato (■), Robertson (▲).

The results of FLiNaK diffusivity measurements carried out across a temperature interval of 730–850 K are given in Table 2 and Figure 3.

The obtained values are close to those of Kato [21] and Roberson (FLiNaK + 0.07% CoF2) [9] (Figure 3). All results fall within an interval of 0.21–0.24 mm^2^/s. The minimal changes in thermal diffusivity with temperature contradict the results of Kato, where the thermal diffusivity rises with temperature. Here, it should be noted that the thermal diffusivity of other salts presented in Kato’s article has no temperature dependence. Typically, the thermal diffusivity data obtained for molten salts demonstrate no temperature dependence or very low negative temperature coefficients [22,23,24].

**Figure 4:**
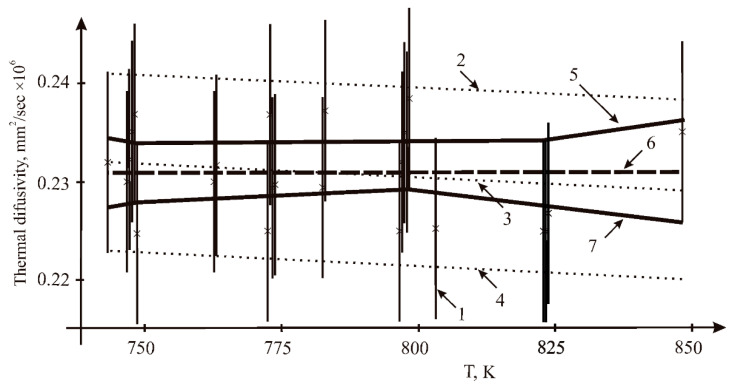
Results of fitting the thermal diffusivity of the sample *n* = 24: 1—measurements (×) and their uncertainty intervals; 2, 4—the range of admissible values by the LSQM (±2σ); 3—the mean value by the LSQM; 5, 7—the interval estimates of the upper and lower limits of the admissible values of the dependencies corridor; 6—the interval estimation of the mean value.

Our data were fitted by two mathematical methods: least square (LSQM) and interval analysis. From the results shown in Figure 4, it can be seen that our results manifest no significant temperature dependence. The bound on the maximum value of the measurement error was equal to 0.0091 mm^2^/s.

In addition to the well-known least square method, the interval approach to data fitting was used. The essential characteristics of this approach follow. Probability characteristics of the measuring error are absent. The following procedures are performed: (1) For each measurement, the interval of uncertainty is calculated using the bound on the maximal measuring error. (2) For each pair of the uncertainty intervals, the possible set of admissible coefficients of the fitting line is constructed. (3) Intersection of all these possible sets of all pairs of the measurement sample provides the sought information set of admissible values for coefficients of the whole sample of measurements with errors. The details of the interval approach can be seen in [25,26].

Resulting values: the least squares evaluation mean value is 0.231 mm^2^/s, σ = 4.55·10^−3^ mm^2^/s. Interval estimation: the average value is 0.232 mm^2^/s; the lower limit—0.229 mm^2^/s; the upper limit—0.234 mm^2^/s.

### 3.2. Heat Capacity

**Figure 5:**
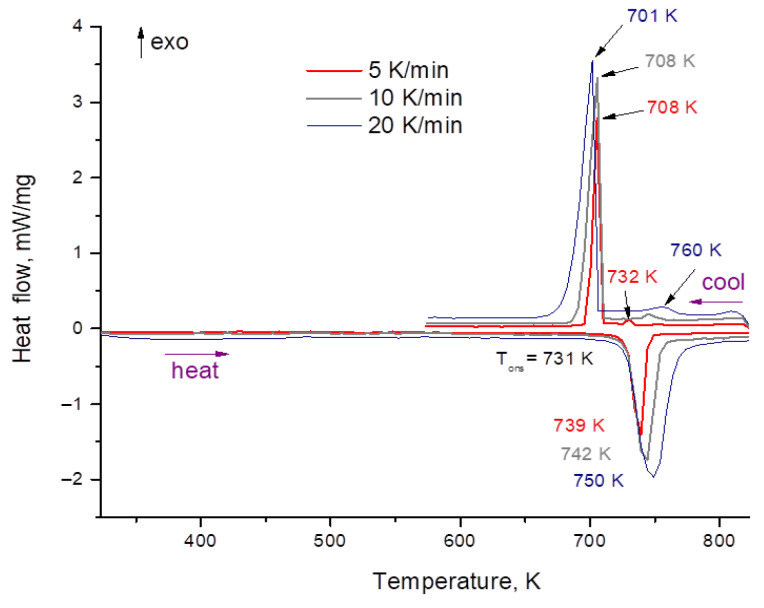
DSC results for FliNaK at different heating rates.

**Figure 6:**
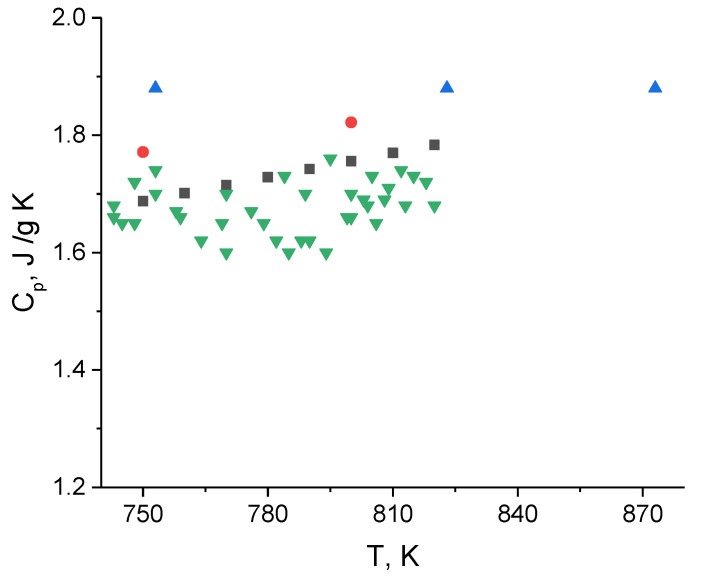
Heat capacity data for FLiNaK: ■—Khokhlov, ●—Janz, ▲—An, ▼—this work.

**Figure 7:**
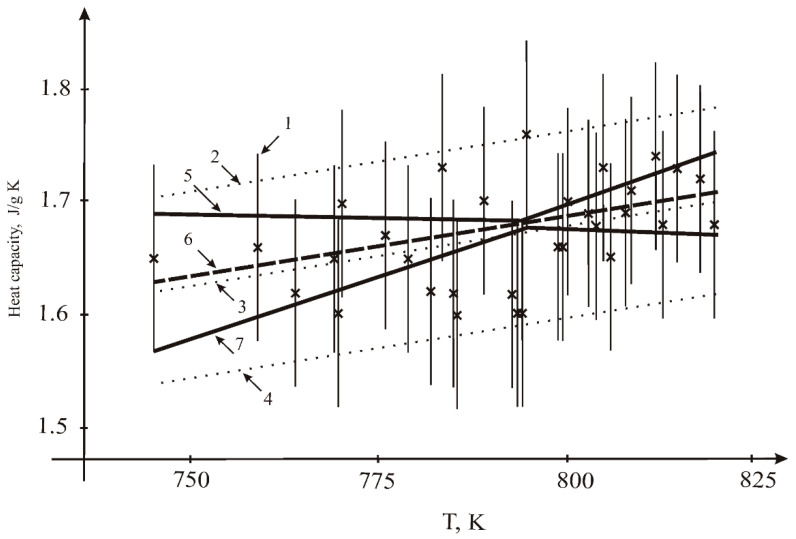
Results for fitting the heat capacity of the sample *n* = 31: 1—measurements (×) and their uncertainty intervals; 2, 4—range of admissible values according to LSQM (±2σ); 3—dependence according to the LSM; 5, 7—interval estimates of the upper and lower limits of the admissible values of the dependencies corridor; 6—interval estimation of linear dependence.

The heat capacity results are given in Table 3. The measurements were carried out at three different heating rates: 5, 10 and 20 K/min. The DSC curves are shown in Figure 5. Overcooling was observed at all heating rates. At cooling, the crystallization takes place at lower temperatures than melting; this does not depend on heating (cooling) rate. There is a small peak corresponding to the melting temperature. At a low cooling rate, the temperature of the small peak corresponds to the melting point on the cooling line. The heat capacity results are independent of the heating rate (Table 3). Our results are close to the data of Khokhlov [5] and Janz [10], but significantly lower than the values obtained by An [6] (Figure 6). The results were treated in the form of linear fit (Figure 7). The bound on the maximum value of the measurement error was equal to 0.083 J/g K. While a small positive temperature dependence was observed for our results, this dependence can be connected with the change of form of the molten sample due to the wetting of walls. Thus, it is possible to consider heat capacity as constant across the small temperature interval (0–100 K above melting point). This value, which was calculated according to the equation of temperature dependence for the melting point temperature, is equal to 1.63 J/g K.

Resulting values of fitting the linear dependence:

- by LSQM: constant A = 0.80536 J/g K; temperature coefficient B = 1.09202 × 10^−3^ J/g K^2^;
- by the interval method: constant A = 0.834 J/g K; temperature coefficient B = 1.096 × 10^−3^ J/g K^2^.

### 3.3. Thermal Conductivity

**Figure 8:**
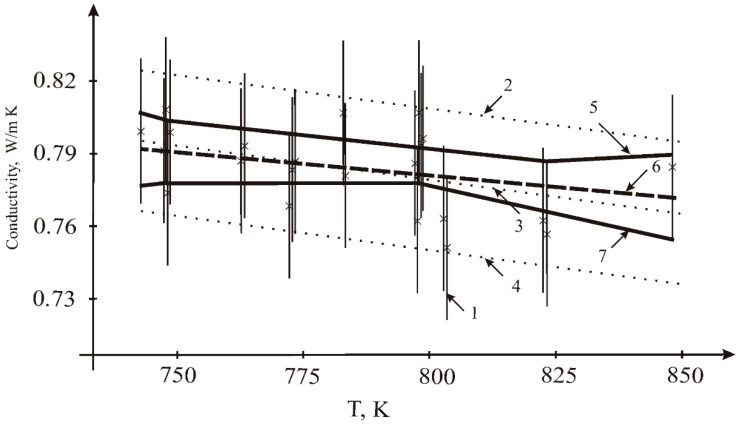
Fitting results for the thermal conductivity of the sample *n* = 22: 1—measurements (×) and their uncertainty intervals; 2, 4—the range of admissible values according to the LSQM (±2σ); 3—the LSQM dependence; 5, 7—interval estimates of the upper and lower limits of admissible values of the dependencies; 6—interval estimation of linear dependence.

The thermal conductivity was calculated on the basis of our data for the thermal diffusivity and heat capacity data, along with results on density from the literature [10]. Our results were treated by linear fitting (Figure 8); the bound on the maximum value of the measurement error was equal 0.083 J/g K.

Resulting values of fitting the linear dependence:

- by LSM: constant *A* = 0.917 Wt/mK; temperature coefficient *B* = −1.869 × 10^−4^ Wt/m K^2^;
- by interval estimation: constant *A* = 0.901 Wt/mK; temperature coefficient *B* = −1.584 × 10^−4^ Wt/m K^2^.

**Figure 9:**
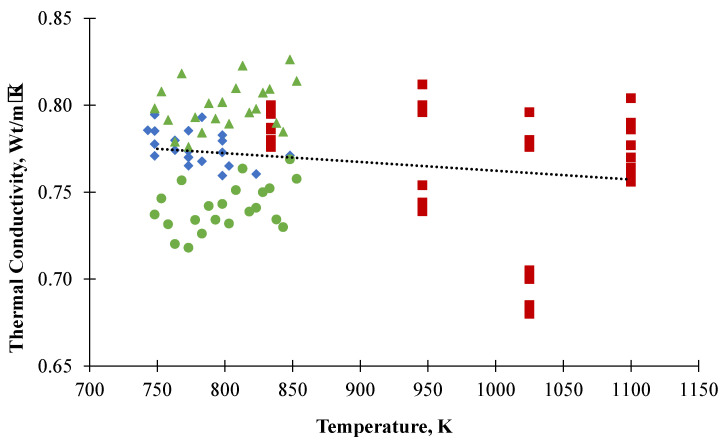
Thermal conductivity results: this work for FLiNaK: (⯁), Gallagher (■), Robertson * (▲), Robertson ** (●). * Calculated with Cp = 1.88 J/g K. ** Calculated with Cp = 1.63 J/g K.

Our results are close to that of Gallagher [8]. The thermal diffusivity data of Roberson [9] can be used for thermal conductivity calculation using different heat capacity results. Being calculated with a heat capacity value of 1.88 J/g K [6], the results will be slightly above those of our data; being calculated with the heat capacity value obtained in this work (1.63 J/g K), the results will be slightly lower than those of our data (Figure 9). The values change insignificantly with temperature (within experimental uncertainty of all data, which is about 8%) and can be presented as a constant for a given temperature interval.

**Figure 10:**
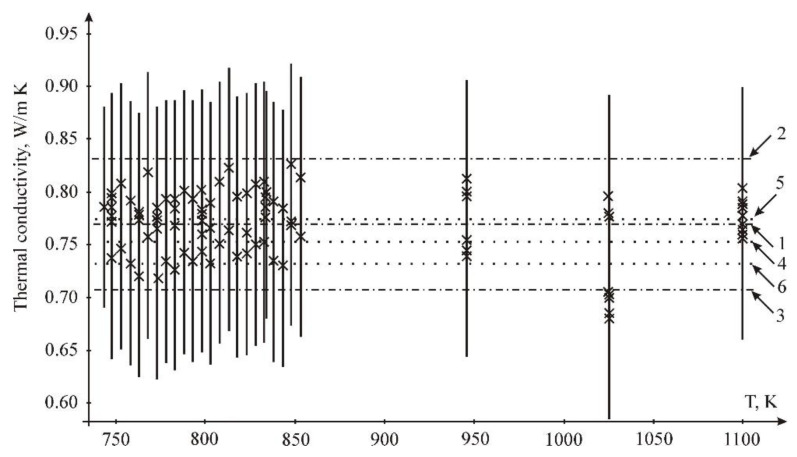
Fitting results for the thermal conductivity of the sample *n* = 96 measurements (×): 1—mean value by LSQM 0.769 W/m K; 2—mean value by LSQM + 2 Sigma W/m K; 3—mean value by LSQM—2 Sigma W/m K W/m K; 4—Interval evaluation 0.753 W/m K; 5—Upper limit of interval evaluation 0.775 W/m K; 6—Lower limit of interval evaluation 0.731 W/m K.

The results of data treatment are given in Figure 10. According to LSQM fitting, the thermal conductivity of molten FLiNaK is 0.77 Wt/m K; according to the interval method, it is 0.75 W/m K

The evaluation is based on three independent investigations performed by three different experimental methods and it means that the obtained values determine the precise interval of thermal conductivity of molten FLiNaK.

The temperature dependence of the thermal conductivity of molten salts is a very questionable issue. Although most of the available data from the literature show a positive temperature coefficient, some of the latest results show a decrease in the thermal conductivity of molten salts with temperature. The question here concerns how to eliminate all of the side mechanisms involved in the heat transfer. The laser flash method is a more effective approach for solving this problem due to the very small size of the sample and concomitant possibility to reduce convective phenomena.

Most theoretical models predict a negative thermal conductivity temperature dependence of molten salts [27,28,29,30,31]. This is due to an increase in the distance between ions, whose length is expected to grow with temperature due to the positive thermal expansion coefficient of molten salts, reducing the number of collisions between ions and decreasing heat transfer. Some thermal conductivity investigations manifest a positive temperature coefficient. Such a result may be due to the complexity of thermal conductivity investigations of molten salts due to high temperatures, along with the instability of molten samples and possible side effects due to the need to measure small temperature differences against the background of high temperature. Methods using relatively large samples such as hot wire, coaxial cylinders and others give positive temperature dependence. Methods dealing with small samples such as laser flash or forced Rayleigh scattering demonstrate negative temperature coefficients. It may be that side effects play a less significant role in small quantities of matter. In general, if a property demonstrates a negative trend in a solid, it continues in the liquid phase (density, speed of sound, etc.). If a property increases with temperature in solid, the same is true of a liquid (e.g., electrical conductivity). Thermal conductivity of fluoride salts in a solid state decrease with temperature and it correlates with thermal expansion [32]. Thermal expansion takes place in molten salts too. Thus, the distances between ions increase with temperature and it decreases heat conduction.

## 4. Conclusions

1. A new experimental technique based on the laser flash method was developed for thermal diffusivity measurements of molten salts;
2. The thermal diffusivity of molten FLiNaK was measured using the laser flash method. It was found to be independent of temperature;
3. The heat capacity was measured by the DSC method. A slight positive temperature dependence was explained by the sample shape change;
4. The thermal conductivity was calculated using the thermal diffusivity, heat capacity and density data from the literature. It was found to decrease with temperature;
5. The results were compared with the latest data from the literature and were found to be in a good agreement.

## Figures and Tables

**Table 1:** Thermophysical data for NaNO_3_-KNO_3_ eutectic mixture.

T, K	α, mm^2^/s	ρ, g/cm^3^ [17]	C_p_, [18] J/g K	λ, Wt/m K	T, K	λ, Wt/m K [19,20]
593	0.150	1.893	1.530	0.435	526	0.468
573	0.151	1.906	1.530	0.440	541	0.465
553	0.153	1.919	1.530	0.449	558	0.458
533	0.159	1.933	1.530	0.471	572	0.454
513	0.157	1.946	1.530	0.469	588	0.448
593	0.159	1.893	1.530	0.460	497	0.462
573	0.153	1.906	1.530	0.446	510	0.460
553	0.157	1.919	1.530	0.461	545	0.457
533	0.158	1.933	1.530	0.467	573	0.446
513	0.156	1.946	1.530	0.465	592	0.446

**Table 2:** Results of thermal diffusivity measurements of FLiNaK.

T, K	α, mm^2^/s	T, K	α, mm^2^/s
823	0.23	783	0.24
798	0.24	743	0.23
773	0.21	803	0.20
798	0.23	783	0.21
773	0.24	763	0.22
748	0.24	743	0.25
823	0.22	803	0.23
798	0.21	783	0.23

**Table 3:** Heat capacity data.

T, K	C_p_, J/g K	T, K	C_p_, J/g K	T, K	C_p_, J/g K
	Exp 1(20)		Exp 2(10)		Exp 3(5)
803	1.69	759	1.66	765	1.6
808	1.69	764	1.62	770	1.6
813	1.68	769	1.65	775	1.58
818	1.72	774	1.65	780	1.6
770	1.7	779	1.65	785	1.62
776	1.67	784	1.73	790	1.76
782	1.62	789	1.7	795	1.7
788	1.62	794	1.6	800	1.73
794	1.6	799	1.66	805	1.72
800	1.66	804	1.68	810	1.73
806	1.65	809	1.71	815	1.68
812	1.74	814	1.77	820	1.67

## Data Availability

The data presented in this study are contained within the article.

## References

[1] Chliatzou C.C., Assael M.J., Antoniadis K.D., Huber M.L., Wakeman W.A. (2018). Reference correlations for thermal conductivity of 13 inorganic molten salts. J. Phys. Chem. Ref. Data.

[2] Redkin A.A., Zaikov Y.P., Tkacheva O.Y., Kumkov S.I. (2016). Molar thermal conductivity of molten salts. Ionics.

[3] Ewing C.T., Spann J.R., Miller R.R. (1962). Radiant transfer of heat in molten inorganic compounds at high temperatures. J. Chem. Eng. Data.

[4] Smirnov M.V., Khokhlov V.A., Filatov E.S. (1987). Thermal conductivity of molten alkali halides and their mixtures. Electrochim. Acta.

[5] Khokhlov V.A., Korzun I.V., Dokutovich V., Filatov E.S. (2011). Heat capacity and thermal conductivity of molten ternary sodium, potassium, and zirconium fluorides mixtures. J. Nucl. Mater..

[6] An X.-H., Cheng J.-H., Yin H.-Q., Xie L.-D., Zhang P. (2015). Thermal conductivity of high temperature fluoride molten salt determined by laser flash technique. Int. J. Heat Mass Transf..

[7] Ueki Y., Fujita N., Yagi J., Shibaraha M., Sagara A. (2017). Thermal conductivity measurement of fluoride molten salt FLiNaK by transient hot-wire method. High Temp.-High Press..

[8] Gallagher R., Birri A., Russell N., Phan A., Gheribi A. (2022). Investigation of the thermal conductivity of molten LiF-NaF-KF with experiments, theory, and equilibrium molecular dynamics. J. Molec. Liq..

[9] Robertson S., Wiser R., Yang W., Kang D., Choi S., Baglietto E., Short M. (2022). The curious temperature dependence of fluoride molten salt thermal conductivity. J. Appl. Phys..

[10] Rogers T.D., Yoko J., Janz G.J. (1982). Fusion Properties and Heat Capacities of the Eutectic LiF-NaF-KF Melt. J. Chem. Eng. Data.

[11] Janz G.J., Tomkins R.P.T. (1981). Physical Properties Data Compilations Relevant to Energy Storage. IV. Molten Salts: Data on Additional Single and Multicomponent Salt Systems.

[12] Cibulková J., Chrenková M., Vasiljev R., Kremenetsky V., Boča M. (2006). Density and Viscosity of the (LiF + NaF + KF)_eut_ (1) + K_2_TaF_7_ (2) + Ta_2_O_5_ (3) Melts. J. Chem. Eng. Data.

[13] Stankus S.V., Savchenko I.V. (2009). Laser flash method for measurement of liquid metals heat transfer coefficients. Thermophys. Aeromech..

[14] Agazhanov A.S., Khairulin A.R., Abdullaev R.N., Stankus S.V. (2020). Thermophysical properties of the liquid eutectic K-Pb alloy. Thermophys. Aeromech..

[15] Agazhanov A.S., Abdullaev R.N., Samoshkin D.A., Stankus S.V. (2021). Coefficients of Heat Transfer for Liquid Alloys of the Rb–Bi System. Russ. J. Phys. Chem. A.

[16] Agazhanov A.S., Khairulin A.R., Abdullaev R.N., Stankus S.V. (2021). Thermophysical Properties of Liquid K–Pb Alloys. J. Eng. Thermophys..

[17] Janz G., Krebs U., Siegenthaler H., Tomkins R. (1972). Molten salts, Volume 3, Nitrates, nitrites and mixtures. J. Phys. Chem. Ref. Data.

[18] Rogers D., Janz G. (1982). Melting-Crystallysation properties of NaNO_3_-KNO_3_, enthalpies and heat capacities. J. Chem. Eng. Data.

[19] DiGuilio R., Teja A.S. (1992). The thermal conductivity of molten NaNO_3_ KNO_3_ eutectic between between 525 and 590 K. Int. J. Thermophys..

[20] Omotani T., Nagasaka Y., Nagashima A. (1982). Measurement of the thermal conductivity of molten KNO_3_ NaNO_3_ mixtures using transient hot wire method with liquid metal in a capillary probe. Int. J. Thermophys..

[21] Kato Y., Furukawa K., Araki N., Kobayasi K. Thermal diffusivity measurement of molten salts by use of a simple ceramic cell. Proceedings of the 8th European Thermophysical Properties Conference.

[22] Nakazawa N., Nagasaka Y., Nagashima A. (1992). Experimental determination of the thermal diffusivity of molten alkali halides by the forced Rayleigh scattering method. II. Molten NaBr, KBr, RbBr, and CsBr. Int. J. Thermophys..

[23] Nakazawa N., Nagasaka Y., Nagashima A. (1992). Experimental determination of the thermal diffusivity of molten alkali halides by the forced Rayleigh scattering method. III. molten NaI, KI, RbI, and CsI. Int. J. Thermophys..

[24] Nakazawa N., Nagasaka Y., Nagashima A. (1992). Experimental determination of the thermal diffusivity of molten alkali halides by the forced Rayleigh scattering method. I Molten LiCl, NaCl, KCl, RbCl, and CsCl. Int. J. Thermophys..

[25] Shary S.P. Finite Dimensional Interval Analysis.

[26] Kumkov S.I., Nikitin V.S., Ostanina T.N., Rudoy V.M. (2020). Interval processing of electrochemical data. J. Comput. Appl. Math..

[27] Gheribi A., Chartrand P. (2016). Thermal conductivity of molten salt mixtures: Theoretical model supported by equilibrium molecular dynamics simulations. J. Chem. Phys..

[28] DiGuilio R., Teja A. (1992). A rough hard-sphere model for the thermal conductivity of molten salts. Int. J. Thermophys..

[29] Lu J., Yang S., Pan G., Ding J., Liu S., Wang W. (2021). Thermal and Transport Properties of MoltenChloride Salts with PolarizationEffect on Microstructure. Energies.

[30] Zakir’yanov B., Tkachev N. (2020). Thermal conductivity of alkali metal chlorides: Calculation with molecular dynamics method. TVT.

[31] Hossain M., Kassaee M., Jeter S., Teja A. (2014). New Model for the Thermal Conductivity of Molten Salts. Int. J. Thermophys..

[32] Popov P.A., Sidorov A.A., Kul’chenkov E.A., Anishchenko A.M., Avetissov I.C., Sorokin N.I., Fedorov P.P. (2017). Thermal conductivity and expansion of PbF_2_ single crystals. Ionics.

